# Divergent Annexin A1 expression in periphery and gut is associated with systemic immune activation and impaired gut immune response during SIV infection

**DOI:** 10.1038/srep31157

**Published:** 2016-08-03

**Authors:** Angela A. S. Sena, Tiffany Glavan, Guochun Jiang, Sumathi Sankaran-Walters, Irina Grishina, Satya Dandekar, Luiz R. Goulart

**Affiliations:** 1 Institute of Genetics and Biochemistry, Federal University of Uberlandia, Uberlandia, MG, Brazil; 2 Department of Medical Microbiology and Immunology, School of Medicine, University of California Davis, Davis, CA, USA

## Abstract

HIV-1 disease progression is paradoxically characterized by systemic chronic immune activation and gut mucosal immune dysfunction, which is not fully defined. Annexin A1 (ANXA1), an inflammation modulator, is a potential link between systemic inflammation and gut immune dysfunction during the simian immunodeficiency virus (SIV) infection. Gene expression of ANXA1 and cytokines were assessed in therapy-naïve rhesus macaques during early and chronic stages of SIV infection and compared with SIV-negative controls. ANXA1 expression was suppressed in the gut but systemically increased during early infection. Conversely, ANXA1 expression increased in both compartments during chronic infection. ANXA1 expression in peripheral blood was positively correlated with HLA-DR+CD4+ and CD8+ T-cell frequencies, and negatively associated with the expression of pro-inflammatory cytokines and CCR5. In contrast, the gut mucosa presented an anergic cytokine profile in relation to ANXA1 expression. *In vitro* stimulations with ANXA1 peptide resulted in decreased inflammatory response in PBMC but increased activation of gut lymphocytes. Our findings suggest that ANXA1 signaling is dysfunctional in SIV infection, and may contribute to chronic inflammation in periphery and with immune dysfunction in the gut mucosa. Thus, ANXA1 signaling may be a novel therapeutic target for the resolution of immune dysfunction in HIV infection.

Human immunodeficiency virus (HIV) infection causes progressive loss of CD4+ T cells in peripheral blood^1^ and systemic chronic immune activation leading to AIDS^2^. In contrast, the virus causes massive loss of CD4+ T cells and immune dysfunction in the gut mucosa leading to the gut barrier disruption and microbial translocation^3^. The gut tissue damage is correlated with HIV progression^4^. Most investigations in HIV research have focused on the mediators of inflammation while regulation and role of anti-inflammatory molecules in HIV pathogenesis has been understudied. Our understanding is limited about molecular networks or specific pathways that may establish a link between the systemic immune activation and gut immune anergy in HIV infection and explain discordance between these two compartments.

We previously found that gene expression of Annexin A1 (ANXA1), an anti-inflammatory molecule was differentially regulated in the gut of therapy-naïve simian immunodeficiency virus (SIV) infected rhesus macaques, an established non-human primate model of AIDS^5^. ANXA1 is a 37 KDa protein known to be a downstream mediator of glucocorticoids, and a highly active mediator of rapid resolution of inflammation^6^,^7^,^8^,^9^. ANXA1 expression is associated with several inflammatory diseases and autoimmune diseases^10^. However, its role in infectious diseases and specifically, in HIV infection is under-investigated. ANXA1-deficient mice showed increased susceptibility to *Mycobacterium tuberculosis*, which was attributed to an impaired dendritic cell efferocytosis and cross-presentation of antigens to pathogen specific CD8+ T cells^11^. The ANXA1 has potential to regulate and link pathways of systemic immune activation while influencing pathways immune anergy in the gut mucosa during inflammatory diseases. Thus, it could potentially contribute to the discordant immune responses between the gut and peripheral blood compartments in HIV infection. Immune activation in HIV and SIV infections is well characterized by an increased expression of known pro-inflammatory markers (TNF-α, IL-6, IL-1β, MIP-1α, MIP-1β and RANTES) in the activated phenotypes of CD4+ and CD8+ T cells, B, NK and monocytes^12^. The gut mucosal disruption in SIV and HIV infections leads to immunological anergy at the mucosal site^13^,^14^,^15^. The ANXA1 has potential to impact this at several levels. First, ANXA1 may function as an anti-inflammatory protein during the acute inflammatory HIV infection, and also exert an immediate inhibitory action on neutrophil migration and monocyte/macrophage and mast cell-mediators production^16^. Second, ANXA1 has been reported to regulate mucosal inflammation, including oral^17^, nasal^18^, lung^19^ and gastrointestinal^20^,^21^ mucosal sites, and may also promote epithelial wound repair^22^. Lastly, the formyl peptide receptors (FPRs) serve as receptors to ANXA1. These FPRs may also function as HIV co-receptors for virus entry^23^, transmission and infection at epithelial surfaces and within tissue reservoirs of HIV-1^24^. Thus, ANXA1 may compete for the same FPR binding site on HIV envelope molecules^25^,^26^.

We sought to investigate changes in ANXA1 expression in both peripheral and gut mucosal compartments during the course of SIV infection and disease progression. Our data showed divergent ANXA1 gene expression patterns in peripheral blood and gut mucosa *in vivo* during primary acute and chronic stages of viral infection, which may be associated with chronic SIV-infection induced immune activation. Further, expression of ANXA1 was negatively correlated with pro-inflammatory cytokines and positively associated with anti-inflammatory response, which was corroborated by exogenous ANXA1 stimulation of peripheral and gut mucosal immune cells, suggesting that the endogenous ANXA1 signaling may be dysfunctional during SIV infection. Collectively, our data suggest that dysfunctional ANXA1 expression and signaling may not only impact the immune activation in periphery, but also may impair the gut immune responses, consequently leading to SIV disease progression.

## Results

### ANXA1 is differentially expressed in PBMC and gut mucosa during SIV infection

**Figure 1:**
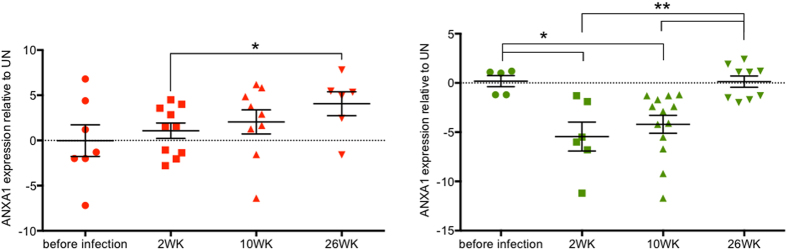
Differential ANXA1 expression in blood and gut throughout the SIV infection. Relative expression of ANXA1 in PBMCs (A) and jejunum biopsy (B) after 2, 10 and 26 weeks of inoculation of Rhesus macaques with pathogenic doses of SIVMac251. Data are presented as fold change of infected versus uninfected animal per dot, with a line representing the mean of the expression’s group. Statistical analysis: p values were obtained on a per group basis (*) using the Mann-Whitney non-parametric test (when comparing different time points).

ANXA1 gene expression was analyzed in both peripheral blood and gut mucosal compartments at pre-infection time point and at 2, 10 and 26 weeks following SIV infection. In the early infection and during transition from acute to chronic stage of infection (2–10wks), the ANXA1 expression was up regulated in peripheral blood of SIV-infected animals (Fig. 1A). In contrast, a striking reduction of ANXA1 expression was seen in the gut mucosa at all time points during SIV infection (fold change average, FC = −4.5) (Fig. 1B), indicating that SIV infection led to differential systemic and mucosal modulation of ANXA1 expression during early stages of infection. During chronic SIV infection (26wk), the ANXA1 expression remained significantly increased in peripheral blood (FC = + 4.2, p < 0.05), as shown in Fig. 1A, and this data was further supported by our previous study of gene expression profiling in SIV infection using microarray analysis^5^. However, in the gut ANXA1 expression was back to normal at 26wk following SIV infection when compared to uninfected controls (FC = +0.5; 26wk vs 2wk, p = 0.009; 26wk vs 10wk, p = 0.002) (Fig. 1B). Together, these data suggested a differential expression profile of ANXA1 between the peripheral blood and the gut both in acute and chronic SIV infections. Dampening the ANXA1 expression in the gut may be a result of the early and rapid loss of CD4+ T cells in this compartment compared to the blood^3^.

### Increased ANXA1 expression in peripheral blood is correlated with immune activation and reservoir establishment

**Figure 2:**
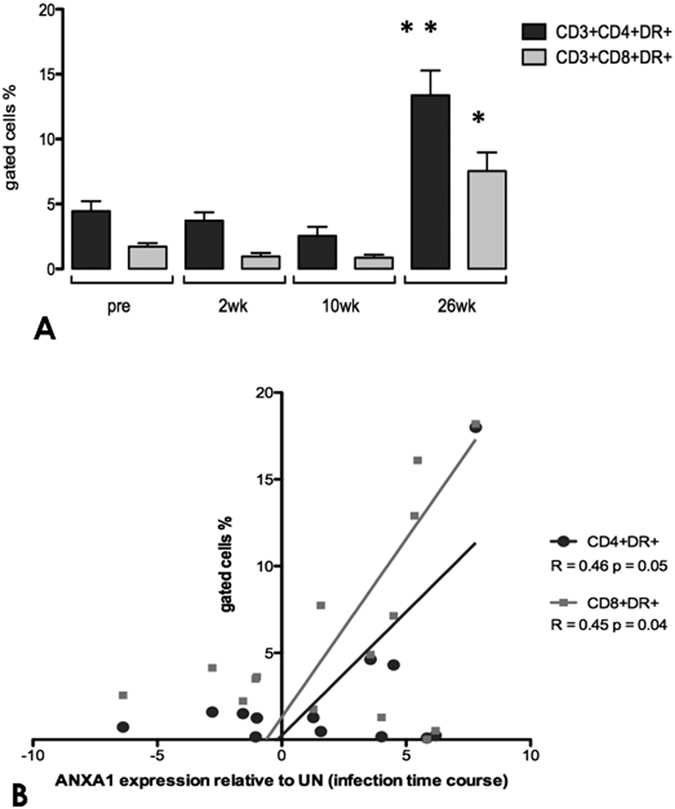
Increased systemic T cell activation correlates with ANXA1 expression. (**A**) Percentage of positive cells for HLA-DR amongst total CD4+ and CD8+ T cells from peripheral blood obtained at the indicated times before and after SIV infection (2, 10, 26 weeks). (**B**) Association of ANXA1 relative expression to the degree of systemic immune activation, as measured by percentage of the activated CD3+ CD4+ DR+ (R = 0.49) and CD3+ CD8+ DR+ (R = 0.47) T cells in the peripheral blood along the course of infection. In (**A**) data are represented as mean ± SEM. Statistical analysis: p values were obtained on a per group basis (*) using the Mann-Whitney nonparametric test (when comparing different time points). *p value < 0.05 and **p value < 0.01 vs early times before and after infection. In (**B**) Spearman’s rank test was used to determine correlations between variables. Both correlations presented p value < 0.05.

It is well established that HLA-DR expression levels on CD4+ T cells are correlated with changes in CD8+ T lymphocytes, plasma HIV antigen loads and the disease progression^27^. Recent study indicated that, among all other immune activation markers, only HLA-DR/DP/DQ(+) expression in CD8+ T cells was associated with persistent HIV infection as well as with HIV reservoir establishment during suppressive ART^28^. To this end, we assessed the percentages of CD4+ and CD8+ T cells expressing HLA-DR by flow cytometry. As expected, in the peripheral blood the number of HLA-DR positive CD4+ and CD8+ T-cells increased during late chronic stage of infection and were coincident with immune activation. Importantly, ANXA1 expression in PBMCs presented significant positive correlation with cellular activation, revealing a similar dynamics of HLA-DR expression in both cell types (Fig. 2A,B), suggesting that expression of ANXA1 may be linked with reservoir establishment *in vivo*.

### The gradual increase of ANXA1 expression during SIV infection may have impaired proper inflammatory responses in the peripheral blood

**Figure 3:**
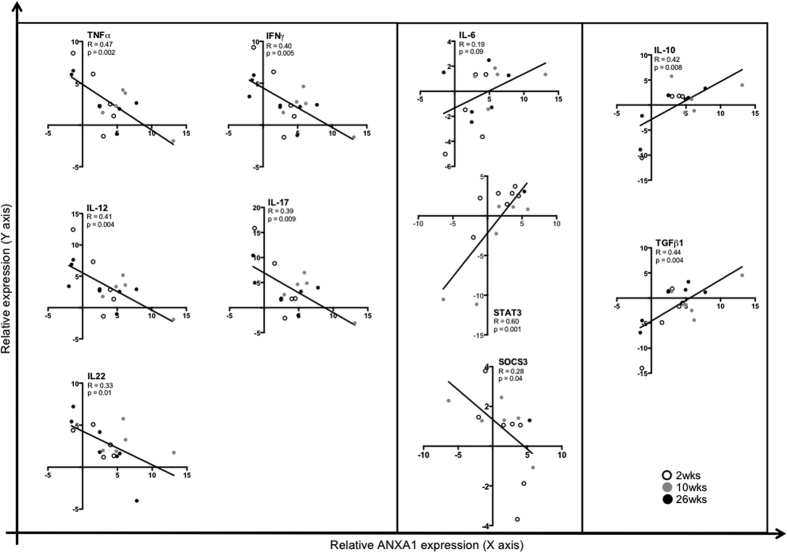
Increased expression of systemic ANXA1 is associated with an anti-inflammatory profile and the IL-6 pathway in the peripheral blood. Plots present tendency lines and the association between ANXA1 and cytokines expression along the SIV infection (2, 10, 26wk), demonstrating the systemic immune activation leading to an anti-inflammatory profile. Spearman’s rank test was used to determine correlations between ANXA1 and cytokines with p value < 0.05.

**Figure 4:**
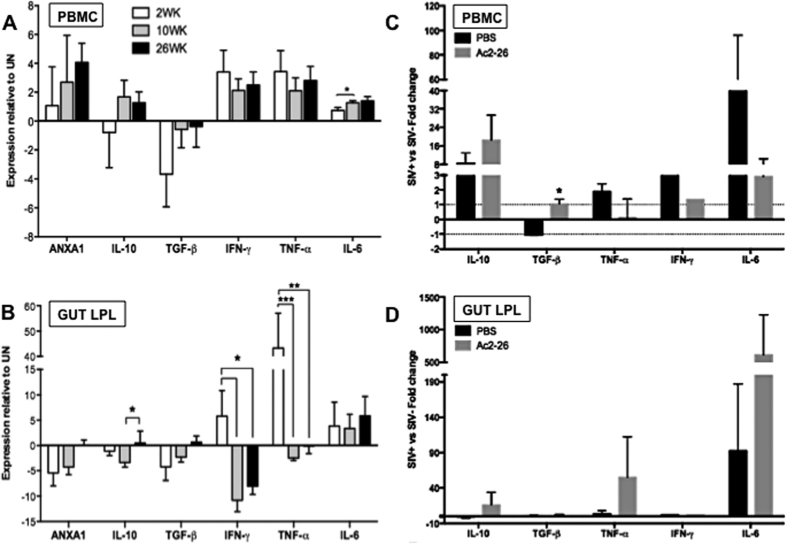
Cytokine expression profiles of PBMCs (**A,C**) and LPLs (**B,D**) from SIV-infected and non-infected animals, according to the endogenous ANXA1 expression (**A,B**) and the exogenous stimuli of the Ac2–26 ANXA1 N-terminal peptide (**C,D**). Relative gene expression of anti-inflammatory (IL-10, TGF-β) and pro-inflammatory (TNF-α, IFN-γ, IL-6) cytokines, in comparison to the endogenous ANXA1 expression profile in both PBMCs (**A**) and LPLs (**B**) along the time course of the SIV infection. Cytokines’ expression profiles were also recorded after Ac2–26 peptide stimuli of both PBMCs (**C**) and LPLs (**D**). Data are represented as mean ± SEM and are presented as fold change of infected versus uninfected animals. Statistical analysis: p values were obtained on a per group basis (*) using the Mann-Whitney nonparametric test, when comparing different time points for (**A,B**) (*p < 0.05 and **p < 0.01 before and after infection), and the Student T test for (**C,D**) (p < 0.05).

Due to the increased ANXA1 gene expression in PBMC and its association with immune activation, we performed additional assessments of specific cytokines and signaling molecules during SIV infection. During early infection (2wk), expression of pro-inflammatory cytokines IFN-γ, TNF-α, IL-17, IL-18, IL-22 (Th1 and Th17 responses) was negatively correlated with ANXA1 expression. At the disease transition (10wk), ANXA1 expression was positively correlated with IL-6/STAT3/SOCS3 signaling, and in the chronic stage (26wk), expression of ANXA1 was positively correlated with anti-inflammatory cytokines IL-10 and TGF-β (Th2/Treg) in the peripheral blood (Figs 3A–C and 4A). Considering these results, we believe that the gradual increase of ANXA1 expression during SIV infection may have impaired proper inflammatory responses in the peripheral blood, facilitating disease progression.

### ANXA1 expression in the gut mucosa parallels with an anti-inflammatory cytokine IL-10 expression

To determine whether the ANXA1 expression correlates with changes in the cytokine expression patterns in the gut mucosa, we measured transcript levels of several cytokines in the gut mucosa during the course of SIV infection. We found a trend of increased expression of pro-inflammatory cytokines (IFN-γ, TNF-α, IL-6) in the gut mucosal compartments during primary SIV infection. Unlike the tendency of remaining elevated in the peripheral blood samples, the expression of IFN-γ and TNF-α were significantly decreased in the gut mucosa during chronic SIV infection. Contrarily, the expression of anti-inflammatory cytokines (IL-10) showed significant increase in the gut when infection transited from acute to chronic infection (Fig. 4B). Divergence in the magnitude of increased pro-inflammatory cytokines in the gut mucosa compared to the peripheral blood during SIV infection may have potentially limited the damage to the tissue microenvironment, and may also have contributed to the incomplete clearance of the virus, which is corroborated by our previous results shown elsewhere^15^.

### Differential cytokine induction between peripheral blood and the gut mucosa stimulated by with Ac2–26 peptide *in vitro*

In order to exert its anti-inflammatory action and impact the disease process, ANXA1 needs to be secreted to induce its signaling pathway^6^. However, based on its divergent expression profile between gut mucosa and PBMC, we hypothesized that the secretion and signaling of ANXA1 may be dysfunctional in both compartments. To investigate exogenous ANXA1 signaling, we stimulated PBMC and LPL cells from chronically SIV-infected rhesus macaques and SIV-negative healthy controls with the N-terminal peptide of ANXA1 (Ac2–26), and cytokine gene expression profiles were assessed. In PBMCs from SIV infected animals, IL-10 and TGF-β levels were induced, while TNF-α, IFN-γ and IL-6 levels were suppressed compared to PBMCs from SIV-negative controls (Fig. 4C). Similarly as in PBMCs, IL-10 was induced by ANXA1 in LPLs from gut mucosa of SIV-infected animals; in contrast to PBMCs, TNF-α and IL-6 were induced following Ac2–26 stimulation (Fig. 4D). These findings reflect the divergent expression of ANXA1 in gut *versus* in PBMCs during SIV infection (Fig. 1), suggesting that SIV infection induced aberrant ANXA1 expression dampens gut immune response, which may be responsible for elevated immune activation in peripheral blood but immune anergy in the gut mucosa (Fig. 2), which is corroborated by our recent findings showing that gut immune responses are massively dampened at chronic stages of the SIV infection^15^.

### Increased ANXA1 expression and systemic activation were negatively correlated with viral load and CD4+ T cell counts

**Figure 5:**
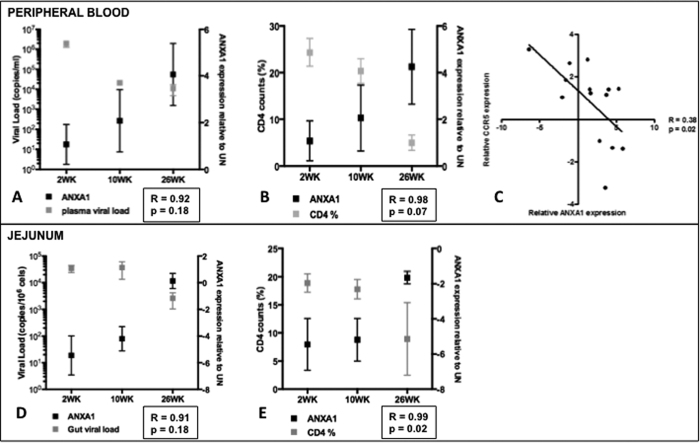
Plasma viral load, CCR5 and CD4 T cell counts correlate to ANXA1 expression in the blood. Kinetics of plasma viral load (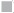) and relative ANXA1 expression (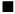) in PBMCs (**A**) and jejunum (**D**) obtained at the indicated times after SIV infection (2, 10, 26 weeks). Kinetics of CD4+ T cell counts (%) and relative ANXA1 expression in the peripheral blood (**B**) and jejunum (**E**) as a function of time after infection. (**C**) Correlation between the relative CCR5 and ANXA1 expression in PBMCs. In (**A,B**) data are presented as mean ± SEM. In (**C**) Spearman’s rank test was used to determine correlation between variables, which presented a moderate positive R = 0.38, p value < 0.05.

The positive correlation between ANXA1 and HLA-DR were discordant from the expected positive association with CD4 counts and viral loads in both compartments, demonstrating contrasting dynamics in peripheral blood and gut mucosal compartments (Fig. 5). In fact, plasma viral load and CD4+ T cell counts declined throughout the infection, accompanied with increased ANXA1 expression in PBMCs and in the gut. Although ANXA1 acts as an anti-inflammatory mediator, the expression levels gradually increased during disease progression (Fig. 5A). These findings were further supported by a negative association between CCR5 and ANXA1 expression in PBMC, showing that the higher ANXA1 levels, the lower the virus co-receptor expression (Fig. 5C), but this was not found in the intestine (data not shown). Despite a trend of decreased viral loads compared to those in the primary viral infection during chronic SIV infection, viral loads persisted (Fig. 5D) and depletion of CD4+ T cells was observed the gut mucosa (Fig. 5E). These changes correlated with increased ANXA1 expression. The increased ANXA1 expression was expected to counteract the chronic activation, which could contribute to the CD4+ T cell survival; however, in the gut ANXA1 expression was dampened, which could explain an inverse correlation between ANXA1 and CD4+ T cell counts, particularly the gut CD4+ T cell depletion during disease progression (Fig. 5B). Taken together, this further supports our hypothesis that the anti-inflammatory ANXA1 signaling is dysfunctional in SIV infection, and aberrant ANXA1 may be associated with systemic immune activation in peripheral blood but immune anergic in the gut.

## Discussion

The systemic activation^29^ and gut immune dysfunction^4^ are two paradoxical hallmarks of SIV infection and AIDS, which have not been well understood. Most of our understanding of chronic immune activation is based on the factors or mechanisms that are pro-inflammatory. However, the balance between the inflammatory and anti-inflammatory mechanisms in the gut mucosa is critical for providing the immune defense while maintaining the integrity and function of the gut microenvironment. We hypothesized that mediators that intersect inflammatory and anti-inflammatory mechanisms may be dysfunctional in the peripheral and gut mucosal compartments. Our investigation identified the ANXA1 protein as a potential mediator in these compartments for many reasons, which are explored herein.

We previously reported that ANXA1 expression was altered in the gut of SIV-infected macaques^5^. This highly active mediator is known to cause rapid resolution of inflammation^6^,^7^,^8^,^9^. However, to exert its anti-inflammatory action, ANXA1 needs to be secreted to trigger its signaling pathway^6^, which is involved in a broad range of biological activities and control of several genes, including transcription factors and signaling molecules^30^,^31^. Signaling through ANXA1 binding to FPR2 receptor involves transient phosphorylation of ERK1, ERK2, AKT and JAK^32^,^33^. The ERK and AKT activation in T cells leads to cell proliferation, whereas ERK and JAK activation leads to a loss of cell adhesion to neutrophils, inhibition of IL-6 signaling and the release of TNF-α from endotoxin-challenged monocytes^34^, resulting in an anti-inflammatory effect. On the other hand, ANXA1 receptor FPR2 also serves as co-receptors for the HIV/SIV gp120^35^ and for a chemotactic peptide of the HIV-1 gp41 ectodomain^24^. Our study is the first to describe changes in the endogenous ANXA1 expression in PBMC and gut mucosa of SIV infected macaques showing a discordant expression between these two compartments. Importantly, striking alterations in expression pattern of major cytokines (IL-10, TGF-β, TNF-α and IL-6) were detected following exogenous stimuli with the ANXA1 N-terminal peptide, Ac2–26, resulting in reduced inflammatory response in the peripheral blood cells while reactivation of the gut immune cell response. These findings led us to propose that endogenous ANXA1 signaling is dysfunctional in both PBMC and gut mucosa during SIV infection, and may be a link between systemic chronic activation and impaired gut immune responses.

Interestingly, we found a positive correlation between HLA-DR+ T cell percentages and ANXA1 levels in PBMC. Up-regulation of ANXA1 expression in T cells following activation and differentiation^7^ is possibly playing a homeostatic regulatory role in mature T cells by modulating the strength of TCR signaling^33^. In pathological conditions, the increased expression of endogenous ANXA1 might contribute to the basal hyper-activated state and to the up-regulation of transcription factors that modulate several inflammatory genes^16^. Consequently, in SIV/HIV infection, the enhanced ANXA1 expression in peripheral blood may provide T cells with positive signals for TCR-induced apoptosis^36^, or could simply be triggered to counteract the active systemic inflammation with its potent anti-inflammatory role. Paradoxically, since HIV replicates most efficiently in activated CD4+ T lymphocytes^37^, the chronic immune activation seems to be important for maintaining virus reservoirs by rendering cells competent for viral replication. Such mechanism is closely related to immune exhaustion, followed by a massive depletion of CD4+ T cells and disease progression^38^,^39^. We have consistently found a negative correlation between ANXA1 transcript levels and CD4+ T cell counts during chronic SIV infection (26 wks), suggest a putative role of ANXA1 in CD4+ T cell apoptosis as a clearance modulator, probably preventing further activation. As HLA-DR was recently identified the only immune activation marker associated with HIV reservoir in the patients under suppressive ART^28^, we only focused on expression of HLA-DR in T cells as an immune activation marker. Our findings that positive correlation of ANXA1 expression with HLD-DR in both CD4+ and CD8+ T cells indicated that ANXA1 could potentially contribute to SIV reservoir establishment (Fig. 2).

We found a significant negative correlation of ANXA1 expression with several pro-inflammatory cytokines and a positive correlation with anti-inflammatory cytokines, such IL-10 and TGF-β, in PBMC of SIV-infected animals, which are associated with immune dysregulation in HIV and considered as negative effects of the infection^40^. But, the low levels of TGF-β and constant release of circulating IL-6 of SIV-infected animals may have compromised the necessary balance between iTregs and Th17 response^41^. It is also possible that the high levels of ANXA1 expression in peripheral blood may have contributed to diminish transcription of pro-inflammatory cytokines. This could promote a transition from Th1 to Th2 T cell response, leading to a defective T-cell activation, and consequently to disease progression. This has been previously corroborated^42^ by the findings from the modulation of ANXA1 in the ANXA1-knockout mouse model^43^, *in vitro* cell culture stimulations with the ANXA1 N-terminal peptide (Ac2–26)^44^, and with glucocorticoids^45^. Our data also revealed a significant positive correlation between ANXA1 and IL-6 expression and its signaling pathway in PBMC, with up-regulation of STAT3 and down-regulation of SOCS3, but not in the gut mucosa. Therefore, IL-6 is evidently a key molecule in SIV infection, since circulating IL-6 levels consistently correlate with residual HIV viremia and markers of immune dysfunction in HIV infection^46^. Some of these findings may be explained by the conversion of IL-6 into an IL-10-like anti-inflammatory response due to the lack of SOCS3 expression in LPS-stimulated macrophages^47^.

The ANXA1 expression was suppressed in the intestine during the initial and transitional stages of SIV infection. Since the major source of ANXA1 in the mucosa are epithelial cells^48^, such down-regulation could be a consequence of the rapid disruption of tissue integrity probably caused by SIV exposure, as previously demonstrated in HIV infection^14^. Conversely, we found in data from previous microarray analyses of Sooty mangabeys that no changes in ANXA1 expression were observed in the gut of this natural hosts of non-pathogenic SIV infections, which do not present severe mucosal CD4 T cell depletion^49^ or tissue damage. Interestingly, in rhesus macaques, the concurrent down-regulation of ANXA1 expression in the gut during acute and chronic infections was followed by an overexpression of TNF-α and IFN-γ. These events may also be linked to tissue injury at early stages due to excessive inflammation without a counteracting anti-inflammatory response, as also evidenced by down-regulation of IL-10 and TGF-β, which as well disrupt the gut regenerative capacity, resulting in loss of mucosal integrity, a mechanism that was not previously identified, but with potential to contribute to the chronic inflammation. This injury may have triggered an enhanced local inflammatory reaction in the acute stage^5^,^50^ followed by an impaired immune response in the chronic stage, probably due to the deprivation of sufficient CD4+ T cells^51^, which has led to the massive dampening of pattern recognition receptors expression detected in the gut mucosa, despite the presence of detectable viral loads^15^, leading to an anergic state in the chronic stage. The major challenge in restoring CD4+ T cells may stem from the inability of the host to repair and regenerate the mucosal damage caused by the virus^50^, which is corroborated by the absence of Annexin A1 in the gut, with direct implications in wound healing, especially in intestinal inflammation and injury^21^. Decreased ANXA1 expression in SIV+ animals during disease progression as described here may induce an inadequate response to bacterial infection^52^, phagocytosis signaling^53^, and Th1-driven responses^33^,^54^.

The negative effect of the endogenous ANXA1 suppression is further evidenced by the compensatory stimuli with exogenous recombinant ANXA1 that resulted in IL-10 release and IL-12 inhibition in LPS-activated macrophages^55^ and also by its putative protective and reparative properties of the intestinal mucosal epithelium against acute injuries^21^. The loss of ANXA1 expression in the gut tissue in SIV infection was associated with chronic infection and an anergic phase^15^, similar to an anti-inflammatory scenario, in which cytokines’ release was maintained at basal levels (TNF-α, IL-10, IFN-γ or TGF-β), except for IL-6 that remained highly expressed, which may have caused a progressive mucosal immune dysfunction, a key feature of the HIV/SIV-associated immune deficiency^4^. The Th17 signaling was also affected as shown by progressive loss of IL-17 and IL-22 as ANXA1 expression increased in the periphery. However, the Th17 response is both permissive and inhibitory of the Th1 response, probably at different phases of the inflammatory process^56^. It has been shown that Th17 cells induced in the presence of IL-1β have a unique messenger RNA profile and an increased capacity to induce inflammation^57^, suggesting that not all Th17 cells are equal. It is interesting to observe that, at early infection, we have seen variable expression of IL-17 coinciding with lower levels of ANXA1, and these results are corroborated by minimal CD4+ Th17 dysregulation in the blood until later stage of HIV infection, while gastrointestinal Th17 depletion was apparent much earlier, along with increased plasma markers of microbial translocation^58^. Meanwhile, lower levels of ANXA1 were maintained in all stages when compared to SIV negative animals, and only reached basal levels in the advanced chronic stage.

Increased plasma IL-6 levels have been associated with HIV-1 disease progression risk^59^, and its increased production in HIV-infected patients at a late stage of the infection did not stimulate HIV replication *in vivo*, but might contribute to metabolic and immunological disturbances associated with the disease^60^. However, the route by which IL-6 comes to be expressed to high levels in blood during HIV infection is still unclear. IL-6 is produced by monocytes and highly induced *in vitro* by HIV infection^61^, and may also be involved in disease progression by promoting T-cell proliferation^62^, because activated T cells replicate HIV in preference to resting T cells^63^. However, LPS-stimulated macrophages infected with HIV were not capable of inducing IL-6 production, and its expression was not increased in lymph node histocultures obtained from HIV-1-infected subjects nor after productive HIV-1 infection of colonic or lymph node histocultures *ex vivo*^59^. Interestingly, IL-6 can reactivate provirus in HIV-1 infected macrophages^64^. This controversial role of IL-6, and divergent behavior between monocytes and macrophages during HIV infection may be due to the persistent defect of infected cells, which is corroborated by our findings of ANXA1 signaling dysfunction in both compartments.

Our studies of *ex vivo* stimulations of PBMCs from SIV infected and SIV-negative macaques with Ac2–26 demonstrated a switch in the immune response of SIV-infected animals from pro-inflammatory to an anti-inflammatory profile, characterized by elevated levels of IL-10 and reduced levels of IL-6, TNF-α and IFN-γ. However, following the divergent behavior between compartments, the gut presented an opposite response, with increased response of IL-10, TNF-α, and IL-6. However, it is important to emphasize that IL-10 levels remained elevated in the blood but presented very low levels in the gut during chronic infection when compared to SIV negative animals. So, the notion that IL-10 signaling presents a negative effect in the disease is still unclear, because its impact in the virus infection *in vivo* is complex and dependent on disease stage^65^. It has also been argued that the interplay of Tregs with monocytes, with upregulation of the IL-10 pathway in the blood might lead to increased disease progression by limiting the host immune responses and impairing pathogen clearance, but it may also function by moderating inflammation, with the benefit of limiting immune-mediated damage to the host^65^. On the other hand, the strong activation of IL-6 in Ac2–26-stimulated LPLs suggests that this cytokine may play a key role in mucosal protection, since its absence was associated with marked infection-induced apoptosis in the colonic epithelium and subsequent ulcerations in a rat model^66^. Interestingly, the massively dampened immune response in the gut during chronic infection^15^ was reactivated by the Ac2–26-stimulated LPLs, as shown by increased levels of TNF-α, and most important, IL-10 and TGF-β expression presented only a small increase, but were maintained in homeostasis, without inhibiting the inflammatory response, which may be considered as beneficial. Therefore, the ability of the exogenous ANXA1 peptide to shape a pro-inflammatory response in the gut and an anti-inflammatory response in PBMC, support the notion that ANXA1 is a homeostatic molecule in both microenvironments and its signaling may be dysfunctional, but the causative mechanism must still be determined.

In progressive HIV/SIV infections, the level of immune activation is a consistent predictor of disease progression as compared to either plasma viral load or peripheral CD4+ T-cell counts^67^. Additionally, our recent findings showed that the gut epithelium was the initial target of viral pathogenesis, as evidenced by impaired expression and disorganization of epithelial tight junction proteins, which were correlated to increased expression of interleukin-1β^68^. In fact, the ANXA1 expression seems to be absent or insufficient, which is responsible to differentially activate several tissue specific cells, which lose the ability of regulating the anti- and pro-inflammatory molecules, such as IL-10, IL-6 and others. Based on our findings of *in vitro* stimulations, we propose that proper ANXA1 expression and signaling may lead to the recovery of the gut immune response and reduce systemic activation. Whether viral gp120 competes for the same ANXA1 receptor and impairs its signaling pathway, or if there are other molecular events modifying the ANXA1 signaling during SIV/HIV infection still remain to be elucidated.

In summary, our data reinforce the importance of understanding the crosstalk between systemic and mucosal immunity through ANXA1 signaling during SIV infection *in vivo*, which apparently acts like a double-edged sword in both processes.

## Materials and Methods

### Animals and Samples

Nonhuman primate studies were performed in accordance with the recommended guidelines of the Public Health Services Policy on Humane Care and Use of Laboratory Animals. Sixty-five colony-bred rhesus macaques (*Macaca mulatta*) were housed at the California National Primate Research Center. Procedures and sample collections were performed according to the protocols approved by the Institutional Animal Care and Use Committee at the University of California, Davis. Anesthetics and analgesics were used during the procedures to minimize pain in animals. Environmental enrichment and feeding were provided in accordance with the recommended guidelines. Animals were euthanized in accordance with the American Veterinary Medical Association Guidelines for the Euthanasia of Animals.

Samples from 12 healthy, SIV-negative animals served as negative controls. Fifty-three animals were intravenously inoculated with animal infectious doses of pathogenic SIVmac251 and were euthanized during primary SIV infection (2week-wk) (n = 16), chronic infection (10wk) (n = 22), and advanced infection with simian AIDS (26wk) (n = 15). A cross-sectional study was carried on with jejunum biopsy samples collected by upper endoscopy and peripheral blood samples by venipuncture in each infection time point. Peripheral blood and intestinal tissue samples were isolated and evaluated as previously described^69^.

### Measurement of Viral Load and Host Gene Expression

Total RNA from PBMC and jejunum biopsy samples were isolated using Qiagen RNeasy Plus Mini Kit in accordance with the manufacturer’s protocol (Qiagen, Valencia, CA). Viral load and host gene expression analyses were performed by quantitative real-time PCR. Briefly, specific primers and probes of the SIV RNA sequence and human cytokines’ gene sequences (ANXA1, CCR5, STAT3, SOCS3, IL10, TGF-β, TNF-α, IFN-γ, IL-6, IL-12, IL-17, IL-18, IL-22), and their reaction conditions are described elsewhere^70^. Fluorescent signals were detected with an ABI Prism 7700 sequence detector (PE Applied Biosystems). Data were captured and analyzed with Sequence Detector software (SDS). Viral copy number in PBMC was determined by plotting CT values against a standard curve. For jejunum samples, relative SIV viral RNA loads were calculated utilizing internal normalization of CT values with housekeeping genes (glyceraldehyde-3-phosphate dehydrogenase, GAPDH; and/or CCR5). For host gene expression, internal normalization of CT values was performed based on GAPDH for each animal, and differential gene expression was calculated by using CT values obtained for each of the SIV-infected animals divided by the mean CT values from four uninfected controls.

### Flow Cytometry

For immunophenotyping of the cells, rhesus-compatible human antibodies were used to detect CD3 (BD Biosciences, clone SP-34-2, San Jose, CA), CD4 (Ebioscience, clone OKT4, San Diego, CA), CD8 (Invitrogen, clone RPA-T8) and HLA-DR (Biolegend, clone L243). To detect cell viability, cells were stained with Amcyan LIVE amine dye (Invitrogen, Carlsbad, CA). Multicolor immunophenotyping of cells was performed on a modified BD LSRII with collection of 1,000,000 events. Flow cytometric analysis was performed using FlowJo version 7.3 (Treestar, Stanford, CA).

### *In vitro* Stimulations with ANXA1 Peptide

Annexin-1N-terminal peptide Ac2–26 (GenScript, USA) was used to stimulate primary peripheral blood mononuclear cells (PBMC) and lamina propria lymphocytes (LPL) from four rhesus macaques with chronic SIV infection and from four uninfected controls. PBMCs and LPLs were isolated as previously described^69^, and incubated at 37 °C for 1 hour in complete RPMI. Cells were centrifuged at 1800 rpm. Aliquots of 1 × 10^5^ cells were added to a 100 μl well, and 100 μM Ac2–26 solution was added to both experimental cells and control medium, in duplicates, followed by incubation at 37 °C for 30 minutes. After stimulations, cells were pipetted into 1.5-ml tubes, centrifuged at 1800 rpm, and medium was discarded. Cells were cryopreserved at −80 °C until RNA extraction. Gene expression was carried out as described before.

### Statistical Analysis

Comparisons between two animal groups or different tissues were performed using two-tailed Mann-Whitney’s U-test or Student’s T test. Pearson’s Correlation Test was used to perform correlation analyses among variables. For correlations, including the PBMC and jejunum tissue viral load, we used the individual viral RNA copy numbers per mL of plasma of each animal at the respective time points when the assay was performed. All statistical analyses were performed by GraphPad Prism 5.0 software (GraphPad Software, San Diego, USA). Data was considered as statistically significant when p < 0.05.

## Additional Information

**How to cite this article**: Sena, A. A. S. *et al*. Divergent Annexin A1 expression in periphery and gut is associated with systemic immune activation and impaired gut immune response during SIV infection. *Sci. Rep.* **6**, 31157; doi: 10.1038/srep31157 (2016).
